# Impact of Silymarin Supplements on Liver Enzyme Levels: A Systematic Review

**DOI:** 10.7759/cureus.47608

**Published:** 2023-10-24

**Authors:** Ernesto Calderon Martinez, Domenica Herrera, Saruveish Mogan, Zainab Hameed, Ayesha Altaf Jangda, Tayyaba J Khan, Palvi Mroke, Samar Sajid, Yash R Shah, Imran Baig

**Affiliations:** 1 Biomedical Informatics, Universidad Nacional Autónoma de México, Mexico City, MEX; 2 Internal Medicine, Pontificia Universidad Católica del Ecuador, Quito, ECU; 3 Faculty of Medicine and Health Sciences, Universiti Malaysia Sarawak (UNIMAS), Kuching, MYS; 4 Internal Medicine, Shifa College of Medicine, Islamabad, PAK; 5 Internal Medicine, Ziauddin University, Karachi, PAK; 6 Medicine, Liaquat University of Medical and Health Sciences, Jamshoro, PAK; 7 Internal Medicine, Caribbean Medical University School of Medicine, Willemstad, CUW; 8 Medicine, Dow University of Health Sciences, Karachi, PAK; 9 Medicine, GMERS (Gujarat Medical Education and Research Society) Medical College and Civil Hospital, Sola, Ahmedabad, IND; 10 Internal Medicine, Houston Methodist Hospital, Houston, USA

**Keywords:** herbal supplements, systematic review, hepatology, liver enzyme levels, sylimarin

## Abstract

Silymarin, extracted from milk thistle (*Silybum marianum*), is esteemed for its antioxidative, anti-inflammatory, and antifibrotic properties, notably within liver-related contexts. Nevertheless, a comprehensive grasp of its effects on liver enzymes remains elusive. This systematic review aims to scrutinize the influence of silymarin supplements on liver enzyme levels, elucidating its potential for hepatoprotection. Following PRISMA 2020 guidelines, we systematically reviewed pertinent studies in PubMed/MEDLINE (Medical Literature Analysis and Retrieval System Online). Our inclusion criteria comprised randomized clinical trials (RCTs) published between 1992 and 2023, accessible in English, with a primary focus on liver enzyme levels. Non-original research, ambiguously defined studies, and those lacking essential data were excluded. Of the 1,707 initially identified articles, 29 RCTs met the inclusion criteria, encompassing 3,846 participants with diverse underlying conditions. Silymarin dosages ranged from 140 mg to 420 mg, administered for various durations. Results revealed that 65.5% of the studies reported reduced liver enzyme levels, 20.7% exhibited no significant change, and 13.8% observed elevated liver enzymes. The systematic review implies a potential advantageous influence of silymarin on liver enzyme levels, indicating its hepatoprotective potential. Nevertheless, outcome disparities may stem from comorbidities, suboptimal doses, and underlying diseases. Notably, silymarin's impact on liver enzymes could be context-dependent, with varying responses among different conditions, with the decrease of liver enzyme levels in patients with non-alcoholic fatty liver disease. Silymarin supplements exhibit potential for hepatoprotection by ameliorating liver enzyme levels across diverse conditions. Further research should ascertain optimal dosages and contexts, accounting for individual patient characteristics and underlying diseases.

## Introduction and background

Milk thistle, also known as *Silybum marianum*, yields a potent extract with significant antioxidant, anti-inflammatory, and antifibrotic properties, commonly referred to as silymarin. This botanical product is employed mainly to treat various hepatic conditions [1,2]. Prior to modern advancements in medicine, silymarin was recognized as a therapeutic bioactive treatment mainly for numerous liver conditions in both European and Asian traditional systems [3]. The therapeutic activity of silymarin manifests through its interaction with various receptors and growth factors, including mitogen-activated protein kinases (MAPKs), mammalian target of rapamycin (mTOR), β-catenin, AKT (A kinase (PRKA) C-terminal domain), apoptotic proteins, and inflammatory cytokine gene expression [3,4]. More specifically, it enhances the expression of superoxide dismutase (SOD) within lymphocytes and erythrocytes, leading to increased levels of glutathione and glutathione peroxidase, thus providing antioxidative stress benefits for patients with chronic liver disease [5,6]. Furthermore, it exhibits a protective property for cell membranes by influencing polymerase I and ribosomal RNA (rRNA) transcription, which in turn reduces or ceases the uptake of toxins, further slowing the progression of liver disease [5]. This systematic review aims to highlight the significant pharmacological and clinical impact of silymarin on liver enzyme levels. However, it primarily focuses on its hepato-protective capabilities, particularly its impact on liver enzyme levels. This review seeks to provide a comprehensive understanding of the effects of silymarin and how to monitor patients undergoing this medication [7].

## Review

Methods

The outcome of this systematic review was to find the effect of silymarin on liver enzyme levels, specifically measures of liver function, as assessed through biochemical markers including alanine aminotransferase (ALT), aspartate aminotransferase (AST), alkaline phosphatase (ALP). Relevant studies published from 1992 to 2023, available in English, were searched in the PubMed/MEDLINE (Medical Literature Analysis and Retrieval System Online) database and reviewed according to the Preferred Reporting Items for Systematic Review and Meta-Analysis (PRISMA) 2020 guidelines [8].

Search Strategy

We searched PubMed/MEDLINE using Medical Subject Headings (MeSH) terms and free-text terms related to our research question (Table 1) on August 8, 2023.

**Table 1:** Search string and number of results The search was conducted on PubMed/MEDLINE (Medical Literature Analysis and Retrieval System Online) on August 8, 2023.

Search	Results
("Silymarin" OR "Silyma*" OR "Silimarin" OR "Silymarin"[Mesh] ) AND ("liver enzyme levels" OR "ALT" OR "AST" OR "ALP" OR "Enzymes"[Mesh] OR "Alanine Transaminase"[Mesh] OR "Aspartate Aminotransferases"[Mesh] OR "Alkaline Phosphatase"[Mesh])	1703

Inclusion and Exclusion Criteria

Inclusion and exclusion criteria were used to select only high-quality studies for analysis. Studies involving the administration of silymarin in adult individuals aged more than 18 years and compared to a placebo or standard treatment were included. Studies that did not relate to the impact of silymarin supplements on liver enzyme levels, as well as those that reported on animal models or did not contain original data, were excluded. Additionally, studies that were not available in full text or could not be obtained through interlibrary loans were excluded. The detailed exclusion and inclusion criteria are given below.

Types of study: We meticulously screened and analyzed semi-randomized clinical trials (RCTs). This systematic review included studies that met the following inclusion criteria: RCTs reporting on the impact of silymarin supplements and assessing liver enzyme levels. We excluded case reports, case series, dissertations, book chapters, protocol articles, reviews, news articles, conference abstracts, letters to the editor, editorials, systematic reviews, meta-analyses, cross-sectional studies, cohort studies, and comment publications. Furthermore, we excluded studies that did not provide a clear description of their operationalization, duplicates, and studies for which we were unable to obtain the necessary data or receive a response from the original author via email.

Types of participants: This study has set specific participant selection criteria, including both genders. The focus was on adult-onset liver enzyme levels. We gave preference to articles that reported enzyme levels in patients with prior or current hepatic diseases, but this was not the exclusive determinant for inclusion, as we also included articles involving other diseases or clinical scenarios that could alter liver enzyme levels such as inflammatory conditions, ischemia, trauma, thyroid disorders, and hemolysis. Studies involving pediatric populations (under 18 years of age) and patients who are unable to ingest the medication for various reasons such as swallowing problems, patients with normal enzyme levels, or patients without a first assessment of liver enzyme levels were excluded.

Types of intervention: To be eligible for inclusion in this study, the selected research must have evaluated the effect of silymarin supplement interventions on liver enzyme levels in adult patients. The interventions could have been oral supplements or any other form of consumption. The control group could have received standard care, alternative intervention, or no intervention. Studies that did not involve the administration of silymarin supplements in any of the subgroups or groups were excluded.

Outcomes: The outcomes to be measured included studies that reported relevant outcomes, specifically changes in levels of liver enzymes such as ALT, AST, and ALP. Studies that did not report relevant outcomes related to liver enzyme levels were excluded.

Selection of Studies

Following an initial screening based on the title and abstract, two reviewers (AAJ and ZH) independently selected trials for inclusion in this review using the predetermined inclusion and exclusion criteria. This search was performed using Rayyan (Rayyan Systems Inc., Cambridge, Massachusetts, United States) to extract relevant data, and duplicates were filtered. Keywords were employed to highlight inclusion and exclusion criteria-related words on Rayyan [9].

Any disagreements about the inclusion of studies were resolved through consensus and consultation with a third review author (ECM). Subsequently, a full-text analysis was conducted, with two reviewers (AAJ and ZH) independently selecting trials for inclusion in this review using predetermined inclusion and exclusion criteria. Disagreements about the inclusion of studies were resolved through consensus and consultation with a third review author (ECM).

Assessment of Risk-of-Bias in Included Studies

We conducted the evaluation of the data using the criteria outlined in the Cochrane Handbook. To assess the quality of studies included in the systematic review, we applied the Cochrane RoB 2.0 tool, which examines potential bias in domains including selection, performance, detection, reporting, attrition, and other sources of bias for RCTs [10]. Two independent reviewers evaluated the risk of bias in each study, taking into account the specific criteria and guidelines provided by the respective tools. Any discrepancies between the reviewers were resolved through discussion or by consulting with a third, blinded reviewer as needed. The methodological components of the trials were assessed as having a low, high, or unclear risk of bias in accordance with the Cochrane Handbook for Systematic Reviews of Interventions [11]. Details of any down- or up-grading of the quality of evidence will be presented in the summary of findings table, providing transparency and explanations for the assessment of bias in each included study.

Results

Study Identification and Selection

**Figure 1:**
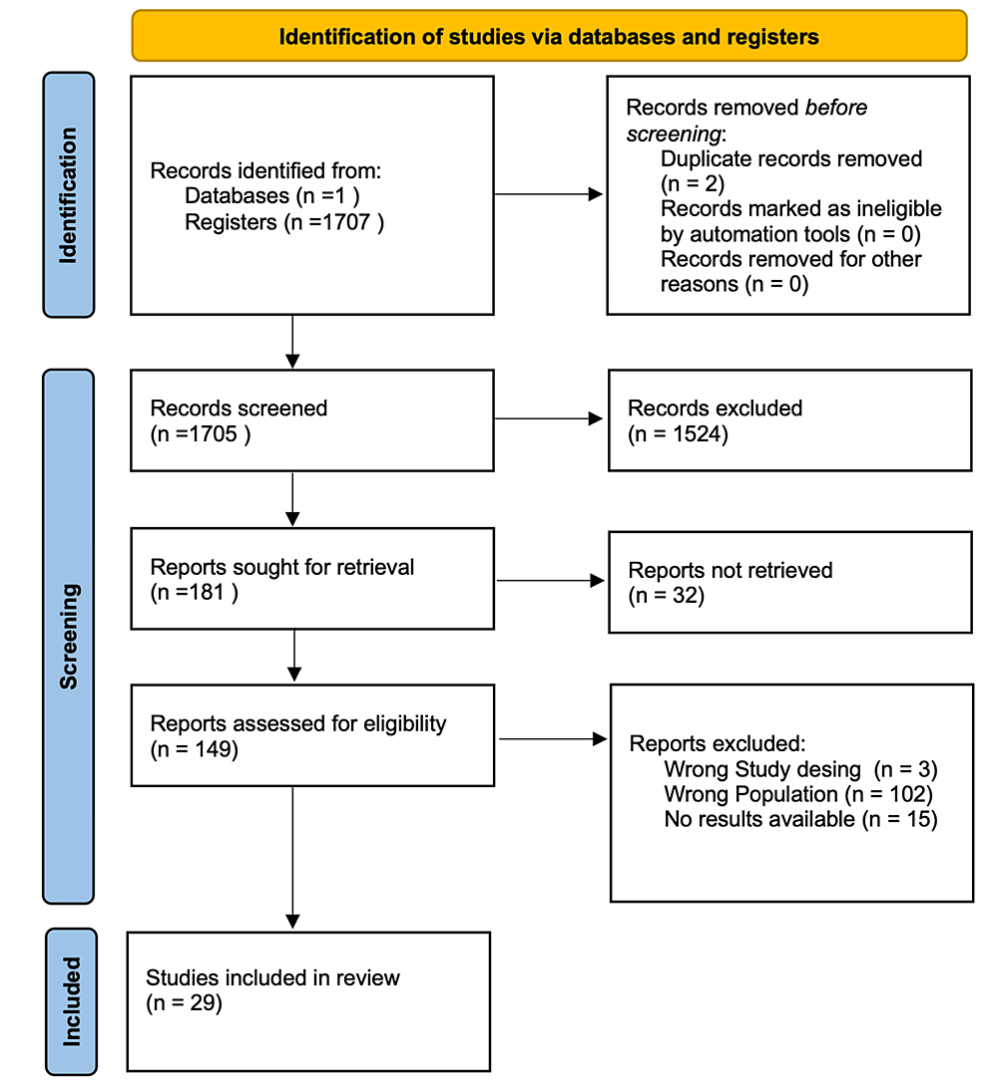
PRISMA flow diagram PRISMA: Preferred Reporting Items for Systematic Reviews and Meta-Analyses

Across the database, we were able to narrow the pool of possible articles down to 1707. After a thorough examination, two duplicate articles were eliminated. A total of 181 publications were selected for further review after an initial screening of titles and abstracts, followed by the retrieval of complete texts. After determining the eligibility and quality of the full-text papers that had been shortlisted, 29 were finally selected for the review process. Figure 1 shows the PRISMA flow chart depicting the study selection procedure. 

The primary outcomes extracted from the finalized research papers were the role of silymarin on blood levels of liver enzymes. The included studies were from a broad geographic range, including countries such as Iran, the United States, Egypt, Spain, Australia, Brazil, Finland, China, Italy, and Pakistan. We reviewed 29 RCTs with a total of 3,846 participants having abnormal liver function tests (LFTs) due to various underlying conditions such as non-alcoholic fatty liver disease (NAFLD), chronic hepatitis C infection, Alzheimer's disease, primary biliary cirrhosis, primary sclerosing cholangitis, trauma, preeclampsia, obesity, multiple sclerosis, hypoxia, isotretinoin treatment, and coronavirus disease 2019 (COVID-19) (Table 2).

**Table 2:** Summary of the articles included for qualitative analysis. RCT: randomized clinical trial; AST: aspartate aminotransferase; ALP: alkaline phosphatase; ALT: alanine aminotransferase; NAFLD: non-alcoholic fatty liver disease

Author, year	Location (Country)	Sample size	Mean age	Duration	Study design	Population characteristics	Intervention with dose	Liver enzyme levels mean, before	Liver enzyme levels mean, after (Result)
Ghalandari et al., 2020 [12]	Iran	60	47±1.73 years	2 Months	RCT	Male patients with Type II Diabetes	Aerobic training+ Silymarin 140 mg X2 daily	AST 24IU/L, ALT 28 IU/L, ALP 200IU/L	There was a reduction in enzyme level: AST 18IU/L, ALT 22IU/L, ALP 160IU/L
Yakoot et al., 2012 [13]	Eygypt	66	48±12 years	6 Months	RCT	Patients with Chronic Hepatitis C Infection	Spirulina 500mg X3 daily vs silymarin 140 mg X3 daily	ALT 75 (43-108), ALP and AST not reported.	AST and ALP not reported, ALT at the end of six months of therapy, silymarin group has minimal effect on liver enzymes as compared to spirulina; ANOVA (F = 8.15, P = 0.006) with mean (SD) = - 23.7 (22.3) and -6.8 (23.2), respectively.
Aller et al., 2015 [14]	Spain	36	47.4±11.2 years	3 Months	RCT	Patients with NAFLD	Silymarin 2 tablets + Vitamin E + Lifestyle modification	AST 35.6 ± 16, ALT 56.4 ± 27, ALP not reported	There was no change in levels. AST 34.6 ± 16, ALT 52.7 ± 26, ALP not reported
Allain et al., 1999 [15]	France	222	F 75±7.03 years, M 74±6.82 years	3 Months	RCT	Patients with Alzheimer’s Disease	Silymarin+tacrine	AST 18 IU/L.	There was an increase in liver enzymes, not statistically significant. AST 22IU/L
Angulo et al., 2000 [16]	USA	27	51.6+/-1.4 years	1 Year	Placebo-controlled trial	Patients with Primary Biliary Cirrhosis With a Suboptimal Response to Ursodeoxycholic Acid	Silymarin, 140 mg, x3 daily	AST. 58 +/- 5 ALT not reported, ALP 897 6+/-84	There was no change in the liver enzyme level. AST56+/-6 ALT ALP 876 +/-95
Angulo et al., 2008 [17]	USA	30	47.3 ± 2.4 years	1 Year	Pilot Study	Patients with Primary Sclerosing Cholangitis	Silymarin, 140 mg x3 daily	AST 116 ± 15, ALT Not reported, ALP 1131 ± 216	There was a reduction in the levels. AST 83 ± 11, ALT not reported, ALP 861 ± 139
Mirzaei et al., 2021 [18]	Iran	107	52.02 ±14.92 years	1 Year	RCT	Blunt abdominal trauma patients in ICU	Silymarin 140mg x3 daily	AST 215.14 ±91.61, ALT 181.84 ±81.59, ALP 272.15 ±44.06	There was a reduction in the liver enzyme level. AST 79.26 ±49.95, ALT 58.75 ±42.10, ALP 154.80 ±40.11
Solhi et al., 2014 [19]	Iran	64	43.6±8.3 years	8 Weeks	Clinical trial	Non-Alcoholic Steatohepatitis Patients	Silymarin 210 mg daily orally for 8 weeks	AST 62.8±10.5, ALT 91.3±21.3, ALP not reported	There was a reduction in the liver enzyme level. AST 30.5±8.2 IU/L, ALT 38.4±11.8 IU/l, ALP not reported
Baghbahadorani et al., 2017 [20]	Iran	Screened with preeclamsia83 eligible 60	20 to 30 years	1.5 Years	RCT	Patients with Severe Preeclampsia	70 mg of Silymarin post delivery at 3 and 24 hours	AST 60, ALT 60	There was a reduction in the liver enzyme level. AST 44, ALT 40
Nehmi-Filho et al., 2022 [21]	Brazil	59	54.10 ± 5.52	6 Months	RCT	Sedentary Volunteers with BMI ≤34.9 kg/m2	2 capsules silymarin contaning nutraceutical supplements	AST 38IU/L ALT 20IU/L	There was an increase, not statistically significant, in the level of liver enzymes. AST 40IU/L, ALT 38 IU/L
Gharagozlooa et al., 2009 [22]	Iran	59	20.2 ± 6.2	3 Months	RCT	B-Thalassemia Major Patients	140 mg x3 daily	AST 28.9 ± 7.6, ALT 32.8 ± 12.7, ALP 549. 5 ± 23.1	There was a reduction in liver enzymes in silymarin group as compared to placebo. AST 42.1 ± 22.3, ALT 42.7 ± 18.6, ALP 459.1 ± 169.8
Gordon et al., 2006 [23]	Australia	24	43±7	12 Weeks	RCT	Patients with Chronic Hepatitis C	*S. marianum* (either 600 mg or 1200 mg)	ALT 100 ± 51, ALP 83 ± 21, AST not reported	There was no change in the liver enzyme level. ALT 86 ± 37, ALP 81 ± 16, AST not reported
Hajiaghamohammadi et al., 2012 [24]	Iran	66	32.62±6.4 years	2 Months	RCT	NAFLD Patients	Silymarin 140 mg daily	AST. 55.31 ± 10.49, ALT.78.18 ± 19.09, ALP no reported	There was a reduction in the liver enzyme level. AST. 37.77 ± 8.78, ALT 53.05 ± 13.99, ALP not reported
Ghiasian et al., 2021 [25]	Iran	48	34.95 ± 11.5 years	6 Months	RCT	MS patients on disease-modifying fingolimod therapy	140 mg silymarin daily	AST 22U/L, ALT 22U/L, ALP 160U/L	There was a reduction in liver enzymes in silymarin group as compared to placebo. AST 22U/L, ALT 18U/L, ALP 190U/L
Jamalian et al., 2020 [26]	Iran	90	56.31 ± 20.01 years.	3 Days	Clinical trial	Patients with Hypoxia	Silymarin 280 mg x3 daily	AST 478.80±113.10, ALT 409.93±80.83, ALP Not reported	There was a reduction in the liver enzyme level. AST 184.17±603.28, ALT 154.77±45.337, ALP Not reported
Kalantaria et al., 2011 [27]	Iran	55	31.8 ± 6.4 years	6 Months	Prospective self-controlled trial	Patients with Chronic Hepatitis C	Silymarin 650 mg daily	AST 99.4 ± 139.7, ALT 108.7 ± 86.6, ALP Not reported	There was a reduction in the liver enzyme level. AST 59.7 ± 64.32, ALT 70.3 ± 57.7, ALP Not reported.
Loguercio et al., 2007 [28]	Italy	85	45 (22–71) years	1 Year	Pilot Study	NAFLD Patients	One pill 94 mg silybin, 194 mg phophatidilcholine, and 90 mg vitamin E.	ALT 79 ± 51, ALP and AST not reported.	There was a reduction in the liver enzyme level. ALT 59 ± 5, ALP. & AST not reported.
Mirhashemi et al., 2022 [29]	Iran	60	38.90 ± 10.28 years	2 Months	RCT	Candidates for bariatric surgery	Silymarin 140 mg X4 daily	AST 35.81 ± 16.57, ALT. 39.64 ± 17.98, ALP not reported	There was a reduction in the liver enzyme level. AST 30.22 ± 15.94, ALT 34.59 ± 17.44, ALP not reported
Curcio et al., 2020 [30]	Italy	81	56.9 Years	3 Months	RCT	NAFLD Patients	Silymarin x2 daily	AST 71.85 IU/L, ALT 81.00 IU/L, ALP 118.46 IU/L	There was a reduction in the liver enzyme level. AST 29.85IU/L, ALT 36.54 IU/L, ALP 82.15IU/L
Mirnezami et al., 2020 [31]	Iran	74	22.19 ± 3.58 years	1 Month	RCT	Acne Vulgaris Patients Taking Isotretinoin.	Silymarin (Livergol) 140 mg for 30 days	AST 21.20 ± 6.47, ALT 19.09 ± 8.67, ALP. 199.12 ± 71.19	There was a reduction in liver enzymes in silymarin group as compared to placebo. AST 19.33 ± 4.55, ALT 16.36 ± 6.33, ALP 193.75 ± 59.99
Fried et al., 2012 [4]	USA	154	54.0 (51.0–58.0) years	6 Months	RCT	Chronic Hepatitis C	Silymarin 420 mg and 700 mg x3 daily in 2 treatment groups	ALT 107.0 (83.0–150.0), ALP and AST not reported.	There was no change in the level of liver enzymes. ALT 104.5 (83.5–151.0), ALP and AST not reported.
El-Kamary et al., 2009 [32]	Egypt	105	29.8± 12.0 years	2 Years	RCT	Patients with Acute Hepatitis	Silymarin 140 mg X3 daily	AST 54 IU/L, ALT 55 IU/L, ALP not reported	There was an increase, not statistically significant, in the level of liver enzymes. AST 6IU/L (16%), ALT 6IU/L (16%), ALP Not reported
Freedman et al., 2011 [33]	USA	1049	50 years	3.5 Years	RCT	Patients with Hepatitis C Antiviral Long-Term Treatment against Cirrhosis (HALT-C)	Took silymarin-containing supplements in past or current status	AST/ALT Ratio 70 (50–102)	AST/ ALT Ratio 71 (49–113), authors suggest no statistical difference
Ahmed et al., 2022 [34]	Pakistan	30	18 - 50 years	2 Months	RCT	Hepatitis C Infection	Silymarin 400mg/day	AST 58 U/L, ALT 78 U/L, ALP 170U/L	There was a reduction in the liver enzyme level. AST 20 U/L, ALT 20 U/L, ALP 80 U/L
Aryan et al., 2022 [35]	Iran	25	49.04 ± 11.14 years	7 Months	RCT	Hospitalized Patients With COVID-19	Silymarin nano micelles 70 mg x3 daily	AST 42.08 ± 3.76, ALT 42.36 ± 4.71, ALP not reported.	There was a reduction in the liver enzyme level. AST 24.20 ± 1.74, ALT 24.52 ± 1.62, ALP not reported.
Tanamly et al., 2004 [36]	Egypt	177	Not reported	12 Months	RCT	Chronic Hepatitis C Infection	Silymarin 140 mg X3 daily	AST, ALT, ALP values not reported.	There is no significant change in liver enzyme levels. Patients who had elevated ALTs receiving silymarin or placebo continued to have elevated ALTs. ALT, AST, and ALP values were not reported.
Cacciapuoti et al., 2013 [37]	Italy	72	44 ± 3.2 years	18 Months	RCT	NAFLD	*Silybum marianum-*containing supplement Epaclin 3.5 g X2 daily	AST 72.39 ± 8.4, ALT 109.48 ± 4.4, ALP not reported	There was a reduction in the liver enzyme level. AST 48.65 ± 3.2, ALT 75.12 ± 3.3, ALP not reported
Hashemi et al., 2009 [38]	Iran	100	39.0 ± 10.70 years	18 Months	RCT	Nonalcoholic Steatohepatitis (NASH)	Silymarin 140mg X2 daily	AST 71.42±66.50 U/L ALT 113.54±50.92 U/L ALP not reported.	There was a reduction in the liver enzyme level. AST 49.66±33.26 U/L ALT 73.14±62.44 U/L ALP not reported.
Deng et al., 2005 [39]	China	96	48.6 years	3 Months	RCT	Patients with NAFLD	Silymarin 200 mg X3 daily	ALT 64.2±11.7 U/L, ALP and AST not reported.	There was a reduction in the liver enzyme level. ALT 48.7±12.4, ALP and AST not reported.

**Figure 2:**
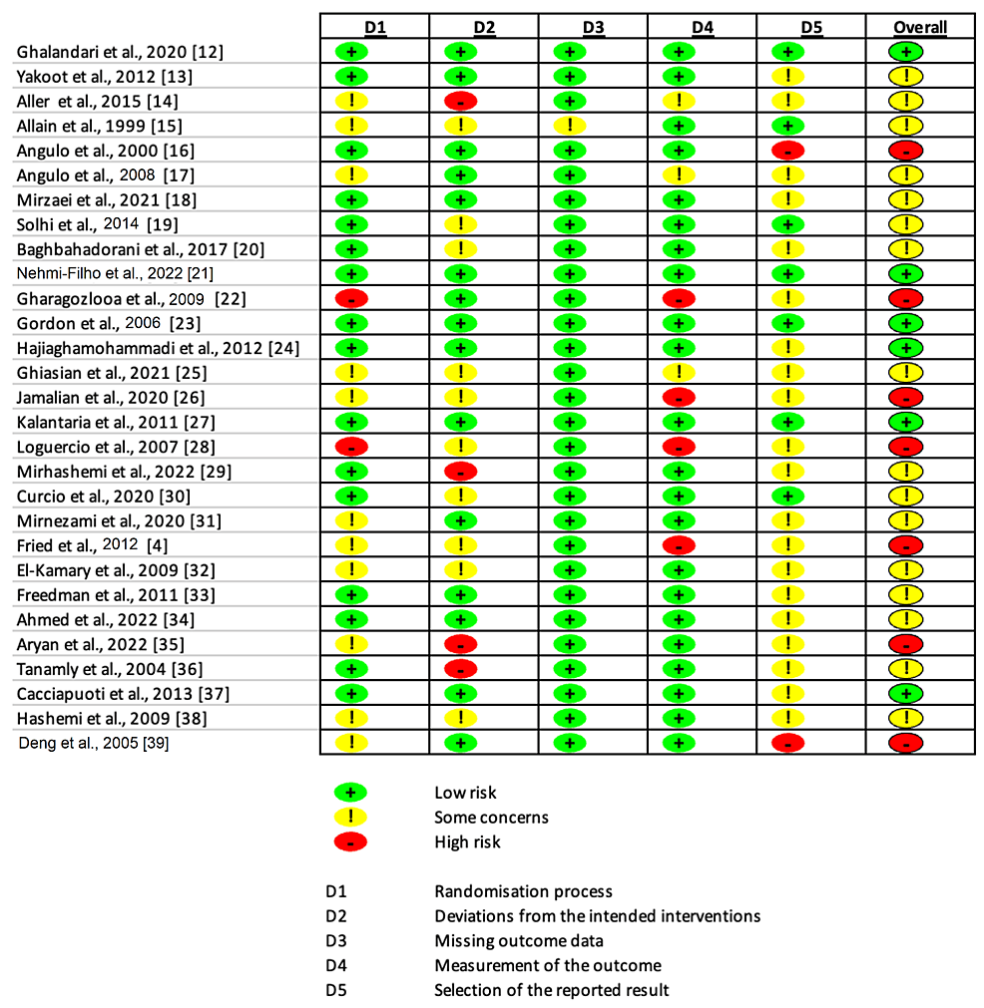
Risk-of-Bias Assessment Assessment made with Risk of Bias 2.0 Tool by Cochrane [10]. References: [4,12-39]

Cochrane's Risk of Bias tool (RoB) was used to assess the quality or risk of bias of the included studies (Figure 2) [10]. The dose of silymarin ranged widely across studies, typically around 140-420 mg, administered multiple times daily. The duration of these studies ranged from a few days to a couple of years. Some studies also combined silymarin with other interventions such as aerobic training, vitamin E, or other drugs. A total of 19 (65.5%) studies reported a decrease in liver enzymes, six (20.7%) indicated no significant change, while four (13.8%) observed an increase in liver enzyme levels upon administration of silymarin. While the results are varied, a significant majority of studies suggest a potential beneficial effect of silymarin on liver enzyme levels.

Discussion 

This systematic review conducted on the impact of silymarin supplements on liver enzyme levels reveals several noteworthy patterns and trends. The liver enzymes utilized as indicators in this study were ALP, ALT, and AST. These specific enzymes were chosen due to their established role as general markers of hepatocellular injury. Raised transaminase levels have been shown to significantly elevate the likelihood of developing end-stage liver disease. As a result, these values serve as a crucial prognostic marker in the context of liver illness [40].

A considerable percentage of the investigations (65.5%) documented a reduction in liver enzyme levels after the administration of silymarin, suggesting a possible advantageous impact on liver well-being. Most notable among these include the studies done by El-Kamary et al. (89.1% and 88.9% reduction in ALT and AST, respectively) [32], Ahmed et al. (74.4% and 65.5% reduction in ALT and AST, respectively) [34], Mirzaei et al. (67.7% and 63.1% reduction in ALT and AST, respectively) [18], and Jamalian et al. (62.3% and 61.5% reduction in ALT and AST, respectively) [26]. In a narrative review, Gillessen and Schmidt examined the use of silymarin as a supportive treatment for liver diseases and reported a similar finding [41]. Their review reported a significant decrease in liver enzyme levels during a treatment period of at least two months with silymarin. Additionally, improvements were observed in liver-related symptoms and overall quality of life. The observed results may be attributed to the hepatoprotective properties of silymarin, which functions as an antioxidant by scavenging free radicals and regulating enzymes involved in the progression of cellular injury, fibrosis, and cirrhosis [42]. The hepatoprotective properties of silymarin make it a dependable therapeutic option in clinical practice, as evidenced by its inclusion in the treatment guidelines for non-alcoholic steatohepatitis in the Asia-Pacific region [43].

In the present review, only a fraction (20.7%) of the participants reported no substantial alteration in liver enzyme levels. One of the primary factors to consider for this finding is the presence of underlying comorbidity. Yakoot and Salem presented a different perspective by comparing the effect of silymarin with spirulina, demonstrating that silymarin had minimal hepatic impact in contrast to spirulina [13]. This comparative analysis highlights the importance of contextualizing silymarin's effects, especially when compared with other potential therapeutic agents. 

This review aimed to evaluate the effects of silymarin on liver enzyme levels in research participants, irrespective of any underlying comorbidities present in the respective studies. The effectiveness of silymarin in decreasing liver enzyme levels is constrained by specific medical conditions. There is a lack of evidence supporting the efficacy of silymarin in patients diagnosed with hepatitis C. A study was conducted to investigate the effects of silymarin on patients with chronic hepatitis C and elevated ALT levels who had previously been treated with interferon [4]. The study was multi-centered, double-blind, and placebo-controlled. A total of 154 patients were enrolled and randomly assigned to receive either silymarin at a dosage of 420 mg or 700 mg three times daily for a duration of 24 weeks. The results of the study indicated that neither dosage of silymarin significantly reduced ALT levels in these patients.

The second element to consider is the mode of administration. According to a meta-analysis conducted by Yang et al. [44]. The administration of silymarin through oral means did not result in any significant variations in serum hepatitis C virus titers, ALT levels, or quality-of-life assessments when compared to the use of a placebo. This analysis included data from five RCTs published up to April 2014, with a total sample size of 389 participants.

The current review further clarified that 13.8% of the studies reported an increase in liver enzyme levels. The observed outcome may be attributed to the administration of a dosage that lacks clinical efficacy. Ensuring a safe and well-tolerated dosage is crucial for achieving optimal outcomes. Nonetheless, this increase was modest when juxtaposed with the reductions observed in other studies. The work done by Nehmi-Filho et al. was noteworthy as it showed an interesting finding of a 90% rise in ALT with a mere 5.3% increase in AST [21]. However, this corresponded to a lowered AST/ALT ratio (AAR), a known biomarker of NAFLD. Based on the findings of Van Wyk and Wink, the administration of silymarin in a dosage range of 200 to 400 mg per day has been deemed efficacious in the treatment of various liver disorders [45]. None of the studies have definitively determined the suitable daily dosage, with only 13.8% of them falling within the range of 200-400 mg. In these trials, it is possible that the liver enzyme levels may have increased due to the progression of the underlying illness and the ineffectiveness of the dosage.

It's crucial to emphasize that most studies have consistently demonstrated a significant decrease in liver enzyme levels among patients with NAFLD. Multiple studies provide substantial evidence supporting this reduction in liver enzyme levels. However, it's important to note that various studies have also indicated a decrease in liver enzyme levels in different contexts, such as hypoxia, COVID-19, obesity, or trauma [18,21,26,35]. Nonetheless, more research is needed to firmly establish the validity of these findings across various diseases.

Limitations

It is vital to comprehend the constraints of our analysis. The review lacked explicit inclusion criteria pertaining to the dosage range, study subjects, mode of administration, and treatment modality, encompassing both monotherapy and polytherapy. The absence of these characteristics introduces a level of complication that could potentially have altered the conclusion of the study. Furthermore, it is important to acknowledge that our review was limited to a single database, confined to papers published in the English language, and concentrated within a specific period. Consequently, it is necessary to recognize that potential bias may exist because of these constraints imposed on our search methodology. There is thus a potential for the omission of older articles and relevant publications in different languages and a possibility that we have failed to include pertinent literature that is presently under review for publication. Although we endeavored to employ a comprehensive search methodology and encompass a diverse range of worldwide evidence, it is possible that there are further studies on this matter within the literature that eluded our attention and analysis.

## Conclusions

This systematic review reveals a compelling trend in the impact of silymarin supplements on liver enzyme levels. The dosage of silymarin emerges as a critical factor influencing its efficacy. This highlights the importance of optimizing silymarin dosing to maximize its hepato-protective properties. Nevertheless, a comprehensive understanding of silymarin's effectiveness in specific types of liver diseases remains a research gap, warranting further exploration.

While numerous studies have explored the bioefficacy and therapeutic benefits of silymarin on LFTs, larger-scale investigations are needed to delve deeper into the underlying pathophysiology and clinical implications associated with silymarin. Such research endeavors would provide a more holistic perspective on its role in liver health and its potential as a therapeutic intervention for a wide range of liver conditions. In summary, silymarin shows promise as a supplement for improving liver enzyme levels, but refining dosing strategies and conducting extensive studies across various liver diseases are essential steps to fully harness its hepato-protective potential.
